# B1a cells play a pathogenic role in the development of autoimmune arthritis

**DOI:** 10.18632/oncotarget.8244

**Published:** 2016-03-21

**Authors:** Jun Deng, Xiaohui Wang, Qian Chen, Xiaoxuan Sun, Fan Xiao, King-Hung Ko, Miaojia Zhang, Liwei Lu

**Affiliations:** ^1^ Department of Pathology and Center of Infection and Immunology, The University of Hong Kong, Hong Kong, China; ^2^ Department of Rheumatology and Immunology, The First Affiliated Hospital of Nanjing Medical University, Jiangsu, China

**Keywords:** B1 cell, receptor activator of nuclear factor kappa-B ligand, collagen-induced arthritis, autoimmune disease, Immunology and Microbiology Section, Immune response, Immunity

## Abstract

Dysregulated functions of B1 cells have been implicated in the disease progression of various autoimmune disorders, but it remains largely unclear whether B1 cells are involved in the pathogenesis of autoimmune arthritis. In this study, we found that peritoneal B1a cells underwent proliferation and migrated to the inflamed joint tissue with upregulated RANKL expression during collagen-induced arthritis (CIA) development in mice. Adoptive transfer of B1a cells exacerbated arthritic severity and joint damage while intraperitoneal depletion of B1 cells ameliorated both arthritic symptoms and joint pathology in CIA mice. In culture, RANKL-expressing B1a cells significantly promoted the expansion of osteoclasts derived from bone marrow cells, which were in accord with the *in vivo* findings of increased osteoclastogenesis in CIA mice transferred with B1a cells. Together, these results have demonstrated a pathogenic role of B1a cells in the development of autoimmune arthritis through RANKL-mediated osteoclastogenesis.

## INTRODUCTION

Recent studies have identified the important contribution of conventional B (B2) cells to the development of autoimmune disease, but our current understanding for the role of B1 cells in autoimmune pathogenesis is still limited [1, 2]. B1 cells are distinguished from conventional B2 cells by their developmental origin, self-renewal capacity, anatomical location and phenotypic feature. B1 cells express high levels of CD19, IgM and CD11b, and can be further divided into CD5^+^ B1a and CD5^−^ B1b subsets. Although B1 cells have been recognized for their protective functions against viruses, bacteria and parasites by producing natural IgM antibodies [3], increasing evidence indicates that B1 cells, especially the highly polyspecific B1a cells, play a role in autoimmune pathogenesis [4]. In mice, B1 cells mainly reside in the peritoneal and pleural cavities, but aberrant B1a cell migration has been found in the pancreas of non-obese diabetic (NOD) mice [5], and in the kidney, thymus and lung of NZBW F1 mice [6-8]. Remarkably, depletion of B1a cells by osmotic pressure ameliorates the development of type I diabetes [5], and reduces renal injury after kidney ischemia or reperfusion [9], indicating the involvement of B1a cells in disease progression. Early reports have found increased CD5^+^ B cells in patients with rheumatoid arthritis (RA) [10, 11], Sjögren's syndrome [12] and systemic lupus erythematosus (SLE) [7, 13, 14]. Recent studies have characterized the human counterpart of murine B1 cells with the phenotype of CD20^+^CD43^+^CD27^+^CD70^−^ present in the umbilical cord and adult peripheral blood [15]. Notably, the frequency of these B1 cells is markedly elevated in SLE patients and correlated with disease pathogenesis and pathopersistence [13].

B1 cells, in particular B1a cells, have been shown to contribute to autoimmune pathogenesis by autoantibody production [7, 8], antigen presentation and activation of CD4 T cells [7, 16], as well as cytokine production [17]. Recent evidence indicates that aberrant B1a cell trafficking is closely correlated with murine lupus progression, a process stimulated by the CXCL13-CXCR5 axis [6]. In addition, B1a cells are found to migrate out of peritoneal cavity (PC) to the spleen, thymus and kidney and produce anti-dsDNA IgG antibodies in NZB/W F1 mice [8]. Furthermore, B1a cells not only activate autoreactive CD4 T cells by its potent antigen-presenting activity, but also preferentially promote Th1 and Th17 cell differentiation and inhibit Treg cell differentiation [16, 18]. Although current studies have revealed the involvement of B1a cells in the pathogenesis of murine lupus and autoimmune diabetes, it is largely unclear whether and how B1a cells participate in the development of autoimmune arthritis.

In this study, we found that peritoneal B1a cells underwent proliferative expansion and subsequently migrated to the inflamed joint tissue with upregulated RANKL expression in mice with collagen-induced arthritis (CIA). Moreover, adoptive transfer of B1a cells markedly exacerbated the arthritic severity and joint damage while intraperitoneal B1 cell depletion ameliorated disease progression in CIA mice. Thus, our results have demonstrated a pathogenic role of B1a cells in the development of autoimmune arthritis.

## RESULTS

### B1a cells undergo proliferative expansion during CIA development

**Figure 1:**
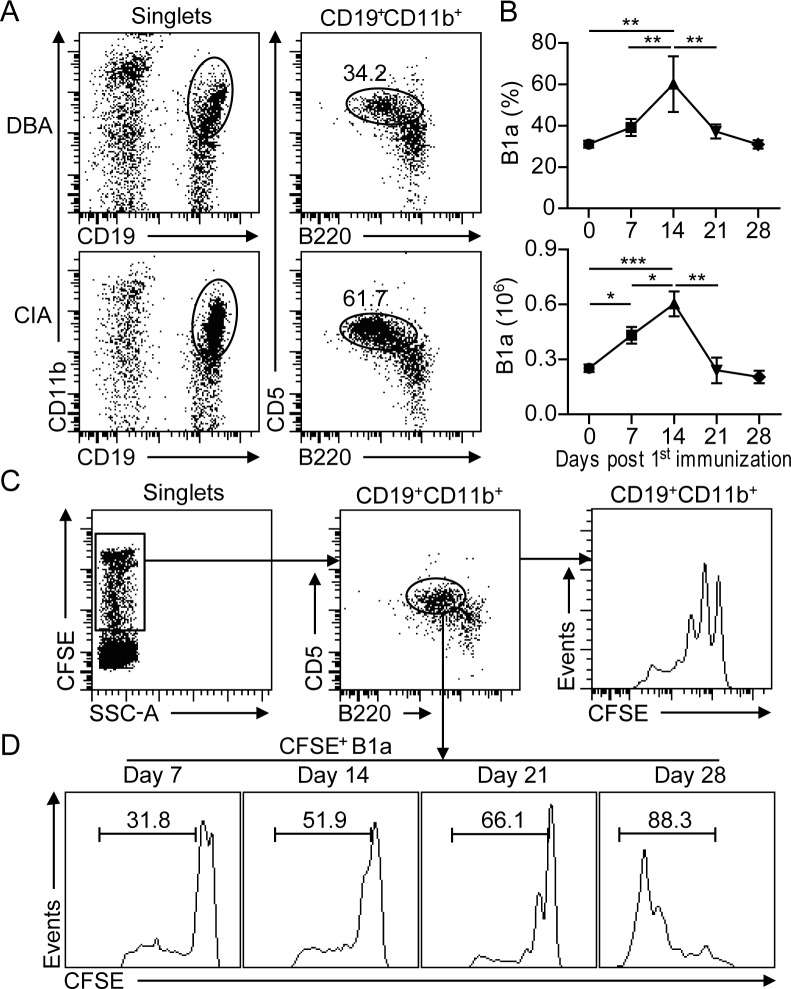
B1a cells undergo proliferative expansion in the peritoneal cavity (PC) during CIA development **A.** Flow cytometric analysis of CD19^+^B220^+^CD11b^+^CD5^+^ B1a cells in the PC of DBA and CIA mice on day 14 post 1^st^ CII-immunization. Flow profiles are representative from five independent experiments. **B.** Frequencies and total numbers of CD19^+^B220^+^CD11b^+^CD5^+^ B1a from the PC of CIA mice were determined by flow cytometry at various time intervals after CII-immunization. Data are derived from five independent experiments and shown as mean ± SD (*, *p* < 0.05, **, *p* < 0.01, ***, *p* < 0.001). **C.** CFSE was intraperitoneally injected into DBA mice and followed by CII immunization for CIA induction. The proliferation of CD19^+^CD11b^+^ B1 cells on day 14 post 1^st^ immunization were determined by flow cytometric analysis. **D.** CFSE-positive CD19^+^B220^+^CD11b^+^CD5^+^ B1a cells in the PC at various time intervals after CII-immunization were measured by flow cytometry. The indicated percentages in C and D are representative of three independent experiments with similar results.

To examine the kinetic changes of peritoneal B1 cells during CIA development, CD19^+^CD11b^+^B220^+^ B1 cells from peritoneal lavage were analyzed by flow cytometry at various time intervals after CII-immunization (Figure 1A). The frequency of CD5^+^ B1a cells started to increase on day 7 post 1^st^ CII-immunization (dpi). Accordingly, total numbers of peritoneal B1a cells were significantly increased on 7 dpi, reached a peak on 14 dpi and followed by a gradual reduction from 21 dpi onward (Figure 1B). To determine whether the increased number of B1a cells was due to proliferative expansion, CFSE was injected into the PC of CII-immunized mice. The proliferative rate of B1a cells was determined by measuring the diluted CFSE fluorescence intensity with flow cytometry (Figure 1C). Remarkably, the proliferative rate of B1a cells was rapidly increased during CIA progression. (Figure 1D). These results indicated that B1a cells underwent proliferative expansion in the peritoneum during the development of CIA.

### B1a cells migrate from peritoneal cavity to the inflamed joint tissue of CIA mice

**Figure 2:**
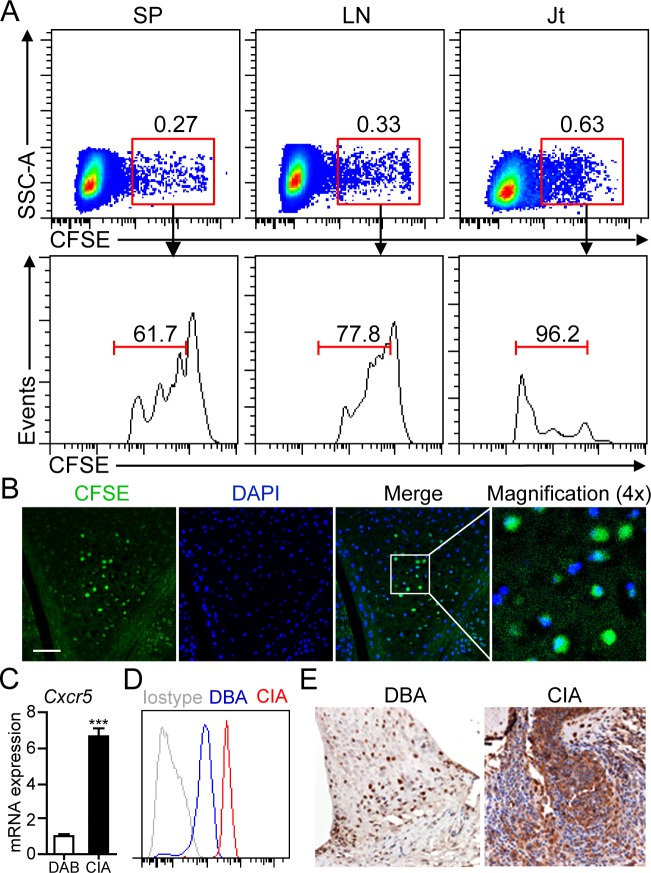
B1a cells migrate from PC to the joint tissue of CIA mice **A.** Sorting-purified peritoneal B1a cells were stained with CFSE and intraperitoneally transferred into DBA mice and followed by CII immunization for CIA induction. On day 17 after cell transfer, CFSE^+^ B1a cells in cell suspensions from the spleen (SP), draining lymph nodes (LN) and joint tissues (Jt) were detected by flow cytometry. Flow profiles are representative from three independent experiments. **B.** CFSE^+^ B1a cells accumulated in the synovium of knee joint of B1a-transferred CIA mice were detected by confocal microscopy (*n* = 5). Scale bar, 50 μm. **C.**, **D.** CXCR5 expression on peritoneal B1a cells from DBA and CIA (14 dpi) mice were measured by q-PCR in C and flow cytometry in D (*n* = 6). Data in C were shown as mean ± SD (***, *p* < 0.001). **E.** CXCL13 expression in the synovium of knee joints of DBA and CIA mice on 17 dpi were measured by immunohistochemistry (IHC) staining. Nucleus was stained with hematoxylin solution. CXCL13-expressing cells are stained an intense brown (Original magnification, ×100) (*n* = 5).

Since gradually decreased numbers of peritoneal B1a cells were observed from 14 dpi onward, we hypothesized that B1a cells may migrate from peritoneal cavity to peripheral lymphoid organs or joint tissue of CIA mice. To test this hypothesis, sorting-purified B1a cells were labeled with CFSE and injected into the PC of DBA mice followed by CII immunization for CIA induction. On day 17 post CFSE^+^ B1a cell transfer, cell suspensions prepared from spleen (SP), draining lymph nodes (LN) and joint tissue were examined by flow cytometry. As expected, a discrete population of CFSE^+^ B1a cells was detected in the SP, LN and joint tissue, respectively (Figure 2A). Notably, CFSE^+^ B1a cells detected in the joint tissue showed the highest proliferative rate when compared with those from SP and LN (Figure 2A). Moreover, CFSE^+^ B1a cells were mainly accumulated in the synovium of knee joint as detected by immunofluorescent microscopy (Figure 2B). Interestingly, we detected markedly increased expression of CXCR5 on peritoneal B1a cells at both mRNA and protein levels from CIA mice when compared with DBA controls (Figure 2C and 2D). In addition, increased CXCL13 expression was detected in the synovial tissue of CIA mice compared with DBA mice (Figure 2E). These findings suggested a possible role of CXCL13-CXCR5 axis in B1a cells migration to the inflamed joint tissue.

### B1a cell transfer or depletion modulates CIA progression

**Figure 3:**
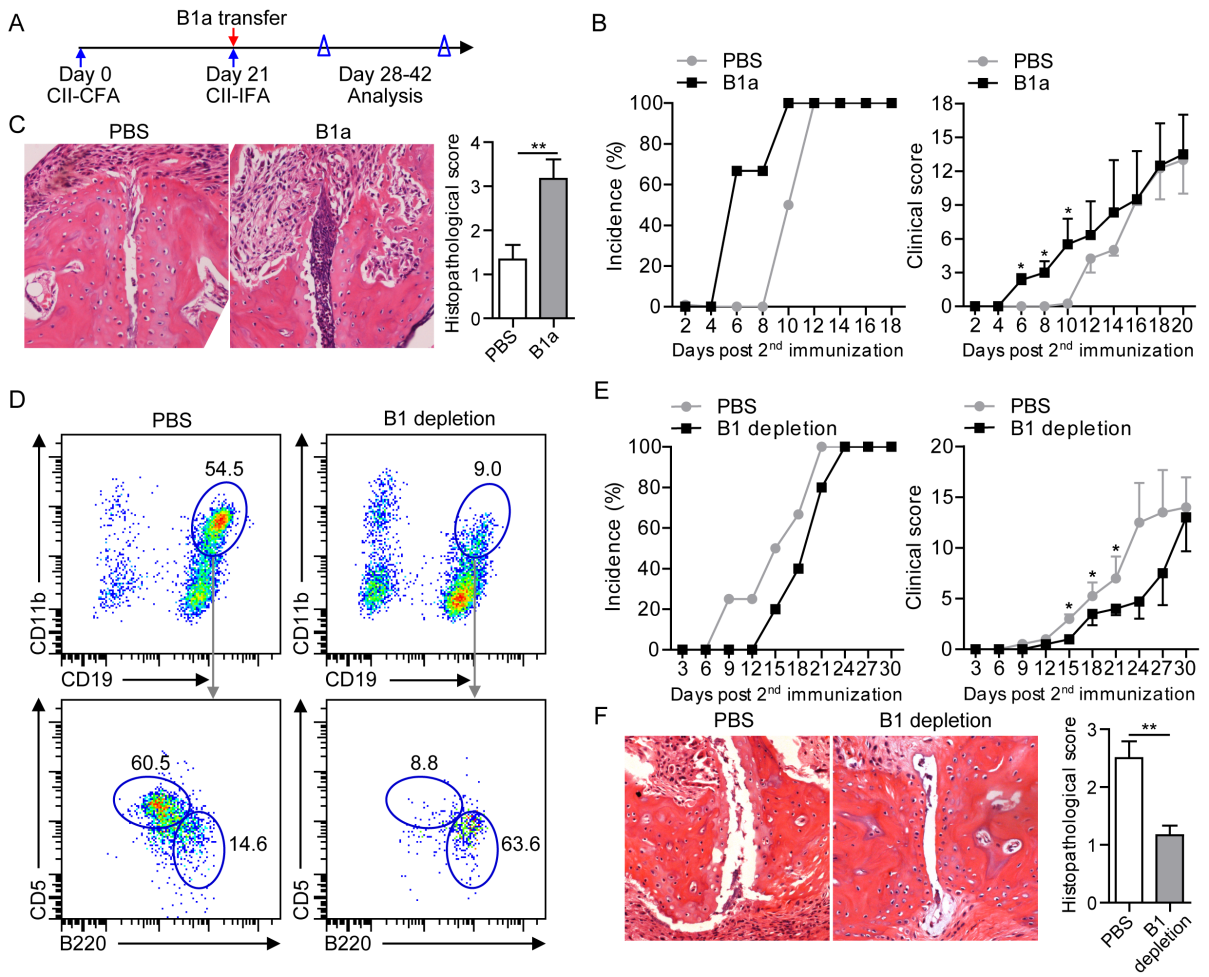
Intraperitoneal transfer or depletion of B1a cells influences CIA progression **A.** Schematic description for CIA induction and time points of B1a cell transfer, and data collection. Sorting-purified peritoneal CD19^+^B220^+^CD11b^+^CD5^+^ B1a cells (1×10^6^) from CII-immunized DBA mice on 7 dpi were intraperitoneally transferred to recipient CIA mice on 21 dpi. Control CIA mice were intraperitoneally injected with the same volume of PBS. Both incidences of arthritis development and clinical scores were analyzed. **B.** Cumulative CIA incidence (100%) and mean clinical score of PBS-treated and B1a cell-transferred CIA mice were calculated daily (five independent experiments, *n* = 7 per group in each experiment). **C.** Histopathologic examination of joint samples from B1a cell-transferred CIA mice and PBS-treated CIA controls in B on day 30 post 1^st^ CII-immunization (H&E staining, original magnification, ×100). Data of histological scores at the right panel are shown as mean ± SD. (*n* = 5 per group, *, *p* < 0.05, **, *p* < 0.01). **D.** B1 cells were depleted by intraperitoneal injection of distilled water every two days for five times from day 17 after 1^st^ CII-immunization (17-25 dpi) whereas control mice were treated with the same volume of PBS injected into the PC of CIA mice. The depletion efficacy of CD19^+^CD11b^+^ B1 cells and CD19^+^CD11b^+^CD5^+^ B1a cells in the PC of PBS-injected and B1 cell-depleted mice were determined by flow cytometry. Data derived from five independent experiments (*n* = 7-10 per group in each experiment) are shown as mean ± SD (*, *p* < 0.05, **, *p* < 0.01, ***, *p* < 0.001). **E.** Both cumulative arthritis incidences and mean clinical scores of PBS-treated CIA mice and B1-depleted CIA mice were calculated from five independent experiments (*n* = 7-10 per group in each experiment). **F.** Histopathologic examination of joint tissue from PBS-injected CIA mice and B1-depleted CIA mice on day 40 post 1^st^ CII-immunization with H&E staining (original magnification, ×100) (*n* = 5 per group). Data are shown as mean ± SD (*, *p* < 0.05).

To determine a role of B1a cells in the development of CIA, sorting-purified peritoneal CD19^+^CD11b^+^CD5^+^ B1a cells were intraperitoneally transferred to 2^nd^ CII-immunized DBA mice on 21 dpi, followed by monitoring the development of arthritic symptoms and histopathology of joint damage (Figure 3A). CIA mice with B1a cell transfer displayed exacerbated arthritis development with an earlier disease onset and higher clinical scores of arthritis symptoms when compared to PBS-treated CIA mice (Figure 3B). Further assessment of synovial hyperplasia, cartilage damage and bone erosion in joint tissue revealed that B1a-transferred CIA mice exhibited more pronounced joint damage with significantly higher histopathological scores when compared with PBS-treated CIA controls (Figure 3C). To further determine whether peritoneal B1 cell depletion may ameliorate the development of CIA, we performed intraperitoneal Milli-Q water injection for B1 cell depletion using a previously reported protocol (Supplementary Figure 1A) [5, 9]. As a result, both frequency and number of peritoneal CD19^+^CD11b^+^ B1 cells were markedly reduced in water-injected mice compared with PBS-injected controls, especially the CD19^+^CD11b^+^B220^+^CD5^+^ B1a cells (Figure 3D and Supplementary Figure 1B). Consistently, total numbers of CD19^+^CD43^+^ B1 and CD19^+^CD43^+^CD5^+^ B1a cells in the joint tissue were drastically decreased in B1-depleted CIA mice when compared to PBS-treated CIA mice (Supplementary Figure 1C). Interestingly, both the incidence of arthritic development and clinical scores of disease severity were significantly lower in B1-depleted CIA mice than PBS-injected CIA controls (Figure 3E). Importantly, B1 cell depletion attenuated the joint pathology with decreased synovial hyperplasia and bone damage in CIA mice (Figure 3F). Together, these results demonstrated a pathogenic role of B1a cells in the development of CIA.

### B1a cells express RANKL in the joint of CIA mice

**Figure 4:**
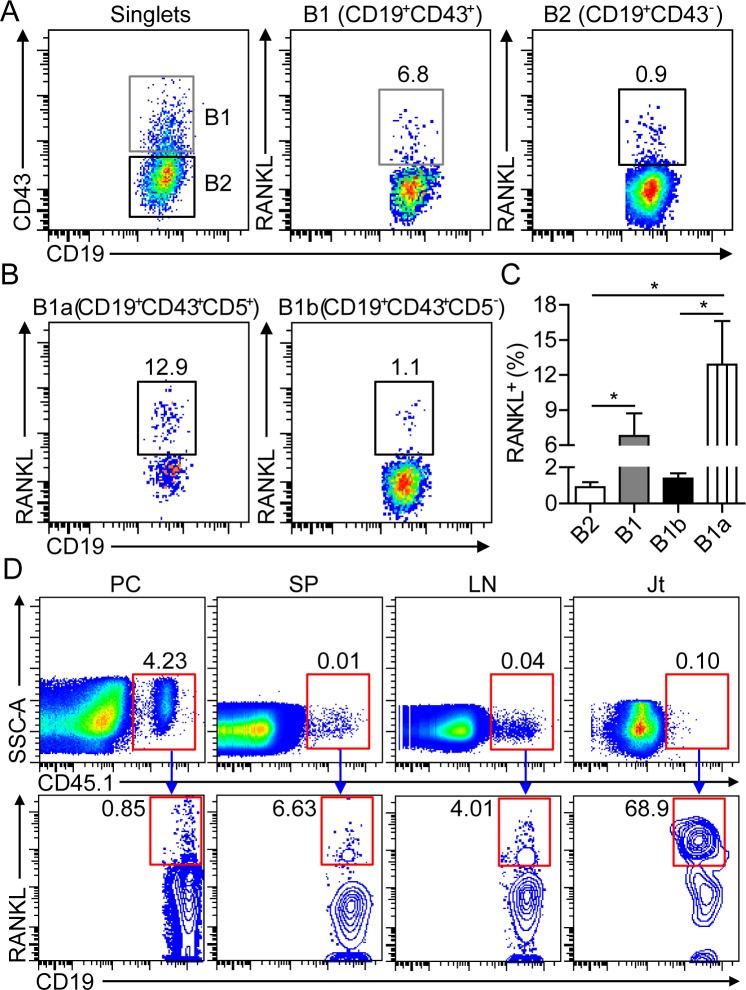
B1a cells express RANKL in the inflamed joint of CIA mice **A.**, **B.** Flow cytometric analysis of RANKL expression on CD19^+^CD43^−^ B2 and CD19^+^CD43^+^ B1 cells in A, CD19^+^CD43^+^CD5^+^ B1a and CD19^+^CD43^+^CD5^−^ B1b cells in B in the inflamed joint of CIA mice on day 30 post 1^st^ CII-immunization. **C.** The frequencies of RANKL^+^ B2, B1, B1a and B1b cells were enumerated from three independent experiments (*n* = 5-7 per group in each experiment). **D.** Peritoneal B1a cells from CD45.1^+^ BoyJ mice were intraperitoneally transferred into CD45.2^+^ C57 mice and followed by CII immunization for CIA induction. On day 17 after cell transfer, RANKL expression on donor CD45.1^+^ B1a cells in the PC, SP, LN and joint tissue (Jt) were detected by flow cytometry. The flow profiles are representative data from three independent experiments with similar results.

In patients with RA, B cells from the synovial cavity were found to express high levels of RANKL [19], but the functional implication of RANKL-producing B cells remained to be addressed. Previous studies showed that B1 cells downregulate CD11b and upregulate CD43 expression upon their egress from peritoneal cavity. CD43 was used as a maker for identifying B1 cells outside of peritoneal and pleural cavities in this study [20-22]. To characterize the functional features of B cells in the inflamed joint of CIA mice, we detected significant RANKL expression on synovial B cells, with a 7-fold higher frequency of RANKL-expressing CD19^+^CD43^+^ B1 than that of CD19^+^CD43^+^ B2 cells (Figure 4A). Moreover, the frequency of RANKL-expressing CD19^+^CD43^+^CD5^+^ B1a cells was approximately 12-fold higher than that of CD43^+^CD5^−^ B1b cells (Figure 4B and 4C). To further confirm the expression of RANKL on migrated B1a cells in the joint tissue, CD45.1^+^ B1a cells from the PC of naive BoyJ mice were intraperitoneally transferred to CD45.2^+^ C57 mice, followed by CII-immunization for CIA induction. Although only less than 1% of sorting-purified CD45.1^+^ B1a cells expressed RANKL prior to transfer, more than 60% of CD45.1^+^ B1a cells expressed high level of RANKL in the joint tissue on day 17 post transfer (Figure 4D). These results demonstrated that B1a cells were the major RANKL-expressing cell subset among synovial B cells in the joint tissue of CIA mice.

### IL-1β and IL-6 induce RANKL expression on B1a cells

**Figure 5:**
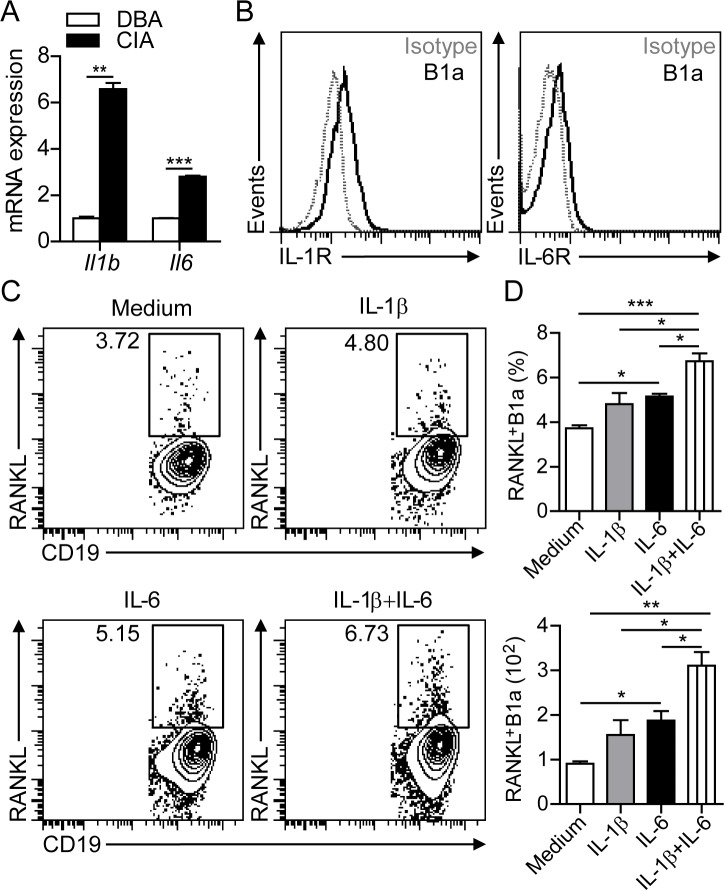
IL-1β and IL-6 induce RANKL expression in B1a cells **A.** Levels of *Il1b* and *Il6* transcripts in the synovium of knee joints of DBA and CIA mice were measured by q-PCR. Data are derived from three independent experiments and shown as mean ± SD. (*n* = 5 per group, **, *p* < 0.01, ***, *p* < 0.001). **B.** IL-1β receptor (IL-1R) and IL-6 receptor (IL-6R) expression on B1a cells from PC of DBA mice were measured by flow cytometric analysis (*n* = 5). **C.** B1a cells from PC of DBA mice were cultured with medium alone, or with medium containing IL-1β, IL-6 and IL-1β + IL-6 for 2 days. RANKL expression on cultured B1a cells were examined by flow cytometric analysis and representative flow cytometric profiles are shown (*n* = 3). **D.** Both frequencies (upper histogram) and total numbers (lower histogram) of cultured RANKL^+^ B1a cells in (C) were calculated from 5 separate experiments. Data are shown as mean ± SD (*, *p* < 0.05, **, *p* < 0.01, ***, *p* < 0.001).

To determine the inflammatory cytokines involved in inducing RANKL expression on B1a cells in the joint, we detected markedly increased levels of IL-1β and IL-6 transcripts in the synovium of CIA mice compared with DBA controls (Figure 5A). In addition, flow cytometric analysis detected the expression of both IL-1β receptor (IL-1R) and IL-6 receptor (IL-6R) on peritoneal B1a cells (Figure 5B). In culture, treatment with either IL-1β or IL-6 significantly increased both the percentage and total numbers of RANKL^+^ B1a cells. Moreover, treatment with IL-1β plus IL-6 showed synergistic effects in inducing RANKL expression on B1a cells (Figure 5C, 5D). In accordance with the *in vitro* studies, flow cytometric analysis revealed the expression of IL-1R and IL-6R on B1a cells in the joint tissue (Supplementary Figure 2A). We also detected upregulated IL-1β and IL-6 in F4/80^+^ cells in joint tissues after CIA induction (Supplementary Figure 2B), suggesting the critical role of IL-1β and IL-6 in the induction of RANKL expression on B1a cells in the inflamed joint. Collectively, these findings showed that proinflammatory cytokines IL-1β and IL-6 could induce RANKL expression on B1a cells.

### RANKL-expressing B1a cells promote osteoclastogenesis from bone marrow cells

**Figure 6:**
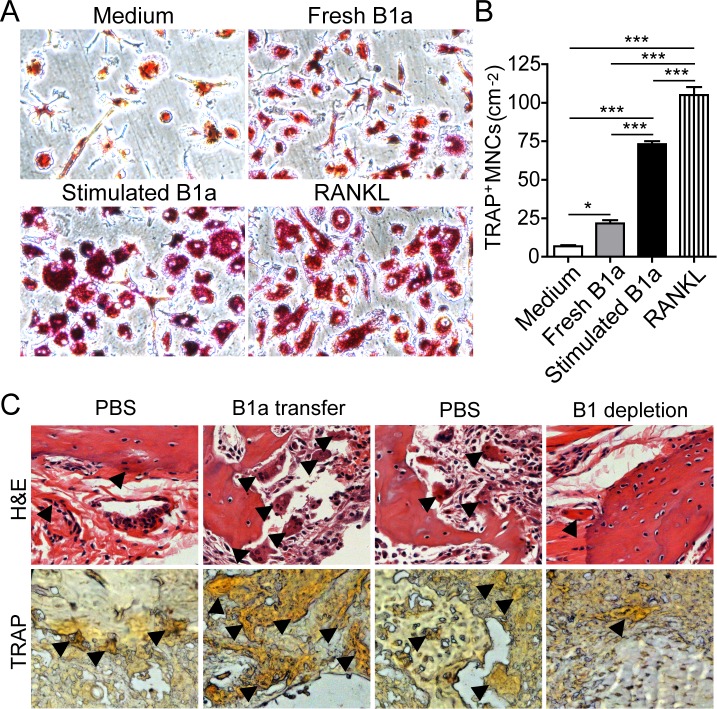
RANKL-expressing B1a cells promote osteoclastogenesis both *in vitro* and *in vivo* **A.** Bone marrow cells (BMCs) were cultured with M-CSF for 3 days and then cocultured with medium only, freshly prepared B1a cells, IL-1β+IL-6 stimulated B1a cells, or medium containing RANKL (20 ng/ml) for 2 days. Osteoclasts were identified by tartrate-resistant acid phosphatase (TRAP) staining (original magnification, ×200). **B.** Cell numbers of TRAP^+^ multinucleated cells (more than three nuclei) were enumerated from five independent experiments. Data are shown as mean ± SD (*n* = 5, *, *p* < 0.05, ***, *p* < 0.001). **C.** Osteoclasts in the joint tissue of B1a transferred CIA mice and PBS-treated CIA mice were identified by H&E in upper panel (black arrow indicated multinucleated cells) and TRAP staining in lower panel (black arrow indicated brown multinucleated cells), respectively (*n* = 5) (original magnification, ×200).

Next, we investigated whether RANKL-expressing B1a cells could induce osteoclastogenesis *in vitro*. Bone marrow cells (BMCs) were first cultured with M-CSF for 3 days. Then, IL-1β- and IL-6-stimulated B1a cells were cocultured with M-CSF-treated BMCs for 5 days. We found that IL-1β- and IL-6-stimulated B1a cells markedly promoted osteoclast differentiation and maturation when compared with freshly isolated B1a cells (Figure 6A and 6B). Consistently, CIA mice transferred with B1a cells exhibited significantly increased numbers of osteoclasts in the joint tissue with exacerbated bone erosion. In contrast, CIA mice with B1 depletion displayed substantially decreased numbers of osteoclasts in joint tissue with ameliorated bone erosion when compared with PBS-treated mice (Figure 6C). These results indicated that synovial RANKL-expressing B1a cells could promote osteoclastogenesis both *in vitro* and *in vivo*.

## DISCUSSION

In this study, we show that peritoneal B1a cells undergo proliferative expansion and migrate to peripheral lymphoid organs and inflamed joint tissue during the development of murine CIA. Moreover, adoptive transfer of B1a cells exacerbates arthritis progression while depletion of peritoneal B1 cells ameliorates disease severity and joint pathology in CIA mice. Inflammatory cytokine IL-1β and IL-6 significantly upregulated RANKL expression on B1a cells which consequently possessed potent capability to induce osteoclastogenesis of bone marrow-derived osteoclasts. Together, these findings demonstrate a pathogenic role of B1a cells in the pathogenesis of autoimmune arthritis.

With unique developmental origin and anatomical location, B1 cells are characterized as a separate lineage of B cells [3]. There is increasing evidence that B1 cells are involved in the development of various autoimmune diseases [4]. In a recent report, Siglec-G deficient mice show an expansion of B1 cell compartment and exhibit increased severity of arthritic symptoms and join inflammation upon CIA induction [23]. Early studies have revealed that aberrant trafficking of B1a cells is correlated with the disease progression of murine lupus [6, 7, 14]. B1 cells have been found to migrate into the thymus and stimulate the proliferation of autoreactive CD4 T cells *via* potent antigen-presenting activity [7]. Here, we show that peritoneal B1a cells during CIA development undergo proliferative expansion before their egress out of PC as revealed by CFSE- live cell staining and flow cytometric analysis. Although it remains currently unclear which factors contribute to the egress of B1 cells from PC during arthritic progression, previous studies have demonstrated that B1 cell migration is activated by signals through toll-like receptors that induce the downregulation of integrins and CD9 [24]. Several endogenous ligands including HMGB1 have been identified to activate TLR4 [25]. Thus, it is reasonable to speculate that endogenous ligands for TLR4 may be involved in promoting B1a cell egress during CIA progression. By intraperitoneally transferring CFSE-labeled B1a cells, we have detected the migration of peritoneal B1a cells to the spleen, draining lymph node and inflamed joint tissue of CIA mice. It is likely that markedly increased levels of CXCL13 expression in the synovial tissue of CIA mice may contribute to the chemotactic attraction of peritoneal B1a cells, a notion supported by previous findings that the CXCL13-CXCR5 axis is responsible for the aberrant trafficking of B1a cells to the kidney in murine lupus [6].

Recent studies suggest that the functional changes of B1a cells may result from their aberrant trafficking to the peripheral lymphoid organs and sites of inflammation during infection and autoimmunity [3, 4]. Previous studies have shown that B1a cells secrete low level of GM-CSF in the PC, but B1a cells proliferate and increase GM-CSF production once they migrate to the spleen or are activated with LPS [26]. Notably, we have identified a population of RANKL-expressing B1a cells that are gradually increased in frequencies from peripheral lymphoid organs to inflamed joint tissue upon their egress from PC. During arthritic progression, activated synoviocytes produce an array of inflammatory cytokines involved in joint pathology in CIA mice. As expected, increased levels of IL-6 and IL-1β transcript expression are detected in inflamed joint tissue where local B1a cells express upregulated IL-1β and IL-6 receptors. In culture, both IL-1β and IL-6 show significant effects on inducing RANKL expression in B1a cells. When CD45.1^+^ B1a cells are intraperitoneally transferred into CD45.2^+^ C57 mice followed by CII-immunization for CIA induction, we have subsequently detected markedly increased frequency of RANKL-expressing CD45.1^+^ B1a cells in the joint tissue, which provide further evidence that local inflammatory cytokines can enhance the functional changes of B1 cells *in situ*.

RANKL is known to be responsible for osteoclast differentiation and activation [27], many studies have demonstrated an important role of RANKL-producing T cells in driving joint pathologies during the development of autoimmune arthritis in mice and RA patients [28]. Although B cells have been recently identified as the main source of RANKL in the synovial fluid of RA patients, it remains to be verified whether B1 cells reside within this RANKL-expressing B cell population [19, 29]. Up to date, the mediators that drive RANKL expression in B cells and the potential role of RANKL-producing B cells in osteoclastogenesis have been poorly investigated [30]. Here, we first show that frequency of RANKL^+^ B1 cells is 7-fold higher than RANKL^+^ B2 cells among synovial B cells from CIA mice. Moreover, RANKL^+^ B1 cells in synovial tissue are predominantly B1a cells. In culture, RANKL^+^ B1a cells can significantly promote osteoclast differentiation from bone marrow cells, which are in accord with *in vivo* findings that CIA mice with B1a cell transfer exhibit increased osteoclast activity with exacerbated bone erosion when compared with control CIA mice. Although our findings suggest a novel role of RANKL-expressing B1a cells in inducing osteoclastogenesis during arthritic progression, further studies on blocking RANKL expression on B1 cells are need to define the role of RANKL expressed on B1a cells in osteoclastogenesis.

There is growing evidence that multifaceted functions of B1 cells other than antibody production are implicated in the development of autoimmune diseases including autoimmune diabetes, lupus and Sjögren's syndrome [6-8, 10-14]. Although the mechanisms by which B1 cells contribute to the pathogenesis of RA remain to be further investigated, our current studies have clearly demonstrated a pathogenic role of B1a cells in the development of murine autoimmune arthritis. Future clinical investigations will provide new insights in understanding the role of B1 cells in the pathogenesis of RA, which may potentially lead to the development of targeting B1 cells as new therapy for RA treatment.

## MATERIALS AND METHODS

### Mice, CIA induction and assessment

DBA/1J, C57BL/6 (CD45.2) and congenic B6.SJL-Ptprc^a^Pepc^b^/BoyJ (CD45.1) mice were purchased from the Jackson Laboratory and maintained in a specific pathogen-free animal facility at the University of Hong Kong with access to food and water *ad libitum*. All animal experiments were approved by the Committee on the Use of Live Animals in Teaching and Research of the University of Hong Kong. Mice were immunized with bovine collagen II (CII) emulsified in Complete Freund Adjuvant and boosted with CII in Incomplete Freund Adjuvant on day 21 after the first immunization for CIA induction while mice immunized with Freund's adjuvant alone served as controls [31]. The incidence and clinical score of CIA were observed daily upon 2^nd^ CII-immunization. For the joint histopathologic examination, paraffin-embedded joint tissue sections were stained with hematoxylin and eosin (H&E) and assessed by a light microscopy (Eclipse E 800, Nikon).

### Cell culture and TRAP staining

Bone marrow cells (BMCs) from DBA mice were cultured with M-CSF (10 ng/ml) in a 96-well plate (1×10^5^ cells per well) for 3 days. Peritoneal B1a cells were cultured in a 96-well plate (1×10^5^) with medium alone, or medium containing IL-1β, IL-6 and IL-1β plus IL-6, respectively, for 2 days. Then, IL-1β plus IL-6 stimulated B1a cells were cocultured with M-CSF-stimulated BMCs for 5 days. Osteoclasts were stained with tartarate-resistant acid phosphatase (TRAP, Sigma) following the manufacturer's instructions, which were identified as TRAP-positive multinucleated cells (MNCs) with the morphology of large multinucleated (more than 2 nuclei per cell) and distinct nucleoli, and coarse azurophilic granules in cytoplasm. The number of TRAP^+^ MNCs was counted at predetermined sites in an area of 1 × 1 mm using a light microscope. Five wells with five sites per well were measured in total.

### Cell sorting and flow cytometric analysis

After preparing single-cell suspensions from the spleen and peritoneal lavage, the cells were stained with FITC-, phycoerythrin (PE)-, phycoerythrin-Cyanin 7 (PE-Cy7)-, allophycocyanin (APC)- and PerCP-Cy5.5- conjugated mAbs against CD19, CD11b, B220, CD5, CD43 and RANKL for cell sorting (FACSAria II cell sorter, BD Biosciences) or flow cytometric analysis (Fortessa, BD Biosciences). The gating strategy used to purify peritoneal CD19^+^B220^+^CD11b^+^CD5^+^ B1a cells were adopted from a previously reported protocol with slight modification [32]. In brief, B1a cells were gated on the singlets determined by FSC-A/FSC-H gating to remove doublets. B1a cells with the purity >97% were used for culture and transfer experiments.

### B1a cell transfer and intraperitoneal B1 cell depletion

Sorting-purified CD19^+^B220^+^CD11b^+^CD5^+^ B1a cells were intraperitoneally transferred into recipient DBA mice on day 21 post 1^st^ CII-immunization whereas control mice were intraperitoneally injected with the same volume of PBS. Using a previously reported protocol for peritoneal B1a cell depletion [5, 9], Milli-Q water (2 ml per mice for each injection) was intraperitoneally injected into mice on days 17-27 post 1^st^ CII-immunization. Control mice were intraperitoneally injected with the same volume of PBS (2 ml per mice for each injection). The depletion efficacy of B1 cells in the peritoneum and joint tissues was determined by flow cytometry.

### CFSE labeling and monitoring of B1 cell migration

Proliferation of peritoneal B1a cell was determined by carboxyfluorescein succinimidyl ester (CFSE) *in situ* detection using a previously reported protocol with slight modification [33]. Briefly, CFSE dissolved in 2 ml of PBS (30 μg/ ml) was intraperitoneally injected into mice on day 21 post 1^st^ CII-immunization, followed by 2^nd^ CII immunization for CIA induction. On day 17 post 2^nd^ CII immunization, CFSE-positive cells in the peritoneal cavity (PC), spleen (SP), draining lymph nodes (LN) and joint tissues (Jt) were determined by flow cytometric analysis. To measure B1a cell migration to the joint tissue, purified B1a cells from the PC of BoyJ (CD45.1^+^) mice were intraperitoneally transferred into C57 (CD45.2^+^) mice followed by CII-immunization for CIA induction. On day 17 post transfer, RANKL expression on CD45.1^+^ B1a cells isolated from joint tissue was examined by flow cytometry.

### Immunofluorescent (IF) and immunohistochemical (IHC) staining

Mouse joint samples were fixed, decalcified, embedded with optimum cutting temperature (OCT, Tissue-Tek) and cut into 6 μm sections. Sections were stained with DAPI to detect CFSE^+^ B1a cells in the synovium of knee joint using a confocal microscopy (LSM 710, Zeiss). CXCL13 expression in the synovium of knee joint was detected by immunohistochemical staining. Paraffin-embedded sections of joint tissue were deparaffinized and rehydrated. After endogenous peroxidase inhibition by 0.5% hydrogen peroxide, sections were blocked with rabbit serum and incubated with biotinylated rabbit anti-mouse CXCL13 (Abcam). CXCL13-expressing cells were identified with StreptABComplex/HRP (Dako) and 3,3-diaminobenzidine tetrahydrochloride solution (Sigma-Aldrich) to develop brown precipitates.

### Statistical analysis

The statistical significance of differences between groups was determined by Student's *t*-tests. Data were analyzed with Prism (version 5.0, GraphPad Software) and shown as mean ± SD. A value of *P* < 0.05 was considered statistically significant.

## SUPPLEMENTARY MATERIAL FIGURES


