# Supplementary material for: B1a cells play a pathogenic role in the development of autoimmune arthritis
Source: Oncotarget. 2016 Mar 21;7(15):19299–311. doi: 10.18632/oncotarget.8244 (PMC4991384; doi:10.18632/oncotarget.8244)
Supplement: Supplementary file 1 [file oncotarget-07-19299-s001.pdf]

B1a cells play a pathogenic role in the development of autoimmune arthritis

Supplementary Material

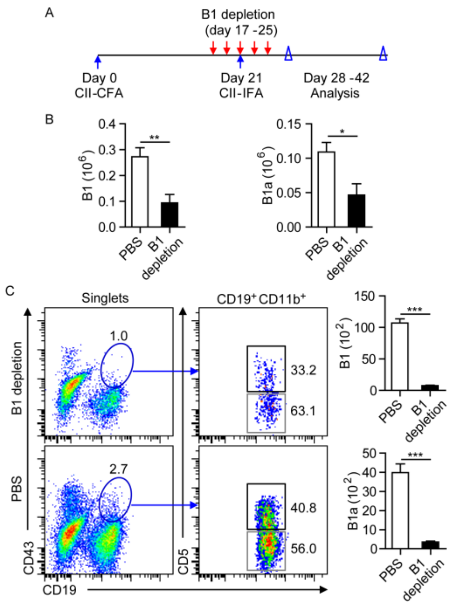

Supplementary Figure 1: Intraperitoneal B1 cell depletion by osmotic pressure in CIA mice. (**A**) Schematic description of intraperitoneal injection of distilled water during CIA induction. B1 cells were depleted by intraperitoneal injection of distilled water every two days for five times from day 17 after 1st CII-immunization (17-25 dpi) whereas control mice were treated with the same volume of PBS injected into the PC of CIA mice. (**B**) The depletion efficacy of CD19^{+}CD11b^{+} B1 cells and CD19^{+}CD11b^{+}CD5^{+} B1a cells in the PC in Figure 3D were enumerated in B**.** (**C**) C19^{+}CD43^{+} B1 and CD19^{+}CD43^{+}CD5^{+} B1a cells in the joint tissues in C of B1 cell-depleted and PBS-injected mice were determined by flow cytometric analysis. The data on the right panel are derived from five independent experiments (n=7-10 per group in each experiment). Data are shown as mean ± SD (*, p<0.05, **, p<0.01, ***, p<0.001).

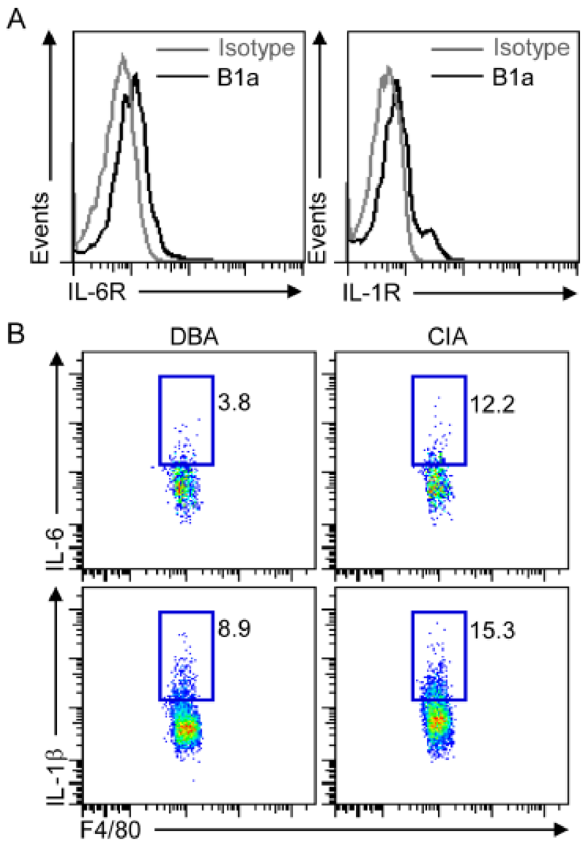

Supplementary Figure 2. IL-1 receptor (IL-1R) and IL-6 receptor (IL-6R) are expressed on B1a cells and IL-1β and IL-6 expression increased in CIA mice. (**A**) IL-1β receptor (IL-1R) and IL-6 receptor (IL-6R) expression on B1a cells from joint of CIA mice at acute stage were measured by flow cytometric analysis (n=5). (**B**) Flow cytometric analysis of IL-1β and IL-6 expression on macrophages in the joints of DBA and CIA mice. Data are derived from three independent experiments and shown as mean ± SD. (n=5 per group, **, p<0.01, ***, p<0.001).
